# Double Assurance of Epidural Space Detection Using Fiberoptics-Based Needle Design and Autofluorescence Technologies for Epidural Blockade in Painless Labor

**DOI:** 10.3390/s18113592

**Published:** 2018-10-23

**Authors:** Cihun-Siyong Alex Gong, Huang-Chang Lee, Yin Chang, Chien-Kun Ting, Po-Hsun Tu

**Affiliations:** 1 Department of Electrical Engineering, School of Electrical and Computer Engineering, College of Engineering, Chang Gung University, Taoyuan 33302, Taiwan; akula.lee@gmail.com; 2 Portable Energy System Group, Green Technology Research Center, College of Engineering, Chang Gung University, Taoyuan 33302, Taiwan; 3 Department of Ophthalmology, Chang Gung Memorial Hospital, Linkou, Taoyuan 33305, Taiwan; 4 Department of Neurosurgery, Chang Gung Memorial Hospital, Linkou, Taoyuan 33302, Taiwan; albert3343@gmail.com; 5 Department of Biomedical Engineering, National Yang-Ming University, Taipei 11221, Taiwan; yc11235813@yahoo.com.tw; 6 Department of Anesthesiology, Taipei Veterans General Hospital, Taipei 11221, Taiwan; 7 School of Medicine, National Yang-Ming University, Taipei 11221, Taiwan; 8 School of Medicine, Chang Gung University, Taoyuan 33302, Taiwan

**Keywords:** epidural space, autofluorescence, optic fiber, ligmentumflavum, double assurance

## Abstract

**Purpose:** Technology of reflectance spectroscopy incorporated with auto-fluorescence spectroscopy were employed to increase the safety of epidural placement in regional anesthesia which is generally used for surgery, epidural anesthesia, post-operative pain control and painless childbirth. **Method:** Ex vivo study of auto-fluorescence spectroscopy was performed for the para-vertebral tissues contained fat, interspinous ligament, supraspinous ligament and ligamentumflavum by multimode microplate reader at wavelength 405 nm for the purpose of tissue differentiation. A specially designed optic-fiber-embedded needle was employed to incorporate with both reflectance and autofluorescence spectroscopies in order to probe the epidural space as double assurance demands. In vivo study was carried out in a Chinese native swine weighted about 30 kg under intubated general anesthesia with ventilation support. The reflective (405 nm) and autofluorescence signals (λ and λ*) were recorded at 5 different sites by an oscilloscope during the needle puncture procedure from skin to epidural space in the back of the swine. **Results:** Study of either autofluorescence spectroscopy for tissue samples or ex vivo needle puncture in porcine trunk tissues indicates that ligmentumflavum has at least 10-fold higher fluorescence intensity than the other tissues. In the in vivo study, ligamentumflavum shows a double-peak character for both reflectance and autofluorescence signals. The epidural space is located right after the drop from the double-peak. Both peaks of reflectance and fluorescence are coincident which ensures that the epidural space is correctly detected. **Conclusions:** The fiber-optical technologies of double-assurance demands for tissue discrimination during epidural needle puncture can not only provide an objective visual information in a real-time fashion but also it can help the operator to achieve much higher success rate in this anesthesia procedure.

## 1. Introduction

Epidural block is a widely used and low-complication-rate procedure which has been applied to many fields of anesthesia such as epidural anesthesia [1,2,3], postoperative analgesia [4] and painless labor [5,6]. It has been reported as an effective clinical technique with lower morbidity and mortality [7] and better analgesia effect as compared to other analgesia methods [8,9].

Anesthesiologists are still looking for a new convenient, real-time and reliable tool for epidural catheter placement to replace the old technique of loss-of-resistance (LOR) which was reported with up to 10% of procedures failure rate due to incorrect epidural space recognition and wrong catheter placement leading to low specificity [10,11,12]. The study in [12] also states that “Mehta and Salmon observed that, in 17% of cases, the needle tip (visualized by the spread of contrast) was positioned either partially or completely outside the epidural canal.”

A two-wavelength fiber-optical method has been developed according to the characteristic reflectance spectra of the tissues during the needle insertion in order to localize the epidural space (ES) [13,14,15]. In our previous animal studies [14], based on the fiber-optical technology [13], EScan be successfully detected according to the characters of reflectance spectra of tissues on the way of needle puncturing. A receiver operating characteristic curve (ROC) was reported up to 0.887 (95% confidence interval (CI) 0.8131–0.9) in terms of the optimal cutoff values for signal amplitudes of 650-nm red laser light [14]. However, if this technology is going to be applied to clinical use, it should provide more convincing evidence in safety aspect.

Autofluorescence is the natural emission of light by cell structures such as mitochondria and lysosomes when they have absorbed light [16]. Collagen and elastin contribute to auto fluorescence because of the intrinsic properties of protein with increasing amount of the amino acids tryptophan, tyrosine and phenylalanine [17]. Double assurance is an idea to this issue if additional technology that can also identify the particular tissue nearby the epidural space. The candidate is ligamentumflavum (LF).

We have observed in our most recent study that LF shows more autofluorescent response to the light of shorter wavelength than the other tissues on the back (para-vertebral tissues) in swine. Longer visible wavelengths do not display noticeable autofluorescence. As a result, in this study, we aim at assessing the feasibility of fluorescent signal as an additional sign for ES detection in addition to reflectance spectrum. Our hypothesis is that these optical techniques can identify and differentiate each tissue layer alone the trajectory of epidural needles in the range of skin to epidural space and assist epidural catheter insertion by a porcine model.

## 2. Materials and Methods

### 2.1. Study Design

This study was approved by the Institutional Animal Care and Use Committee of Taipei Veterans General Hospital. The study was conducted in two phases- ex vivo and in vivo. We conducted the first phase of our optical study in ex vivo porcine tissues. In the second part of our study, we applied information from the ex vivo analysis to our anesthetized porcine model. We analyzed the optical signal in two different applicable wavelengths in the reflective spectra. Wavelengths that provided accurate information on the optical characteristics of the individual tissue in both the ex vivo and in vivo phases of our studies were selected. Technical details are needed regarding the fiber optic diameter and the needle size as well. We used a 17-gauge needle where the contained fiber optic is with a diameter of 405 μm.

In the experiments, there was one pig of averaged 30 kg in weight for studies. The pig was intubated after inducing general anesthesia and mechanically ventilated. In addition, the pig was placed in the left lateral position under the epidural needle puncturing. The needle was inserted ten times for each of the experiments and the resulting numerical value associated with each experiment was an average from ten-time recorded data. The conventional LOR technique was also used to double confirm the effectiveness of the proposed technique. The placement of needle tip was also confirmed by both ultrasound (Vivid e; GE Healthcare, London, UK) and radiography (KXO-50R; Toshiba, Tokyo, Japan) using 5 mL of the contrast agent ioxitalamic acid. The pig was euthanized after the procedures. We used the same probe throughout.

The studies of both phases adopted the same calibration procedure for the devices involved in the experimental setup. All the instrumental parameters including the wavelengths, bandwidths and detector gain were carefully set with varying degrees of repeatability as well as accuracy. The excitation beam intensity was monitored in order to correct the measured fluorescence intensity as a result of the light source intensity wavelength dependency and excitation wavelength selector transmittance. Application of needle is distance traced and therefore all the obtained data were being reconstructed for double confirmation and validation for its efficacy. The velocity of the needle placement has been kept extremely slow to get appropriate results without the influence by needle movement related to the fluorescence. The fluorescence can be a very good tool as the needle advancing is less affected by blood flow.

### 2.2. Phase One—Ex Vivo Studies

#### 2.2.1. Study Case 1

**Figure 1:**
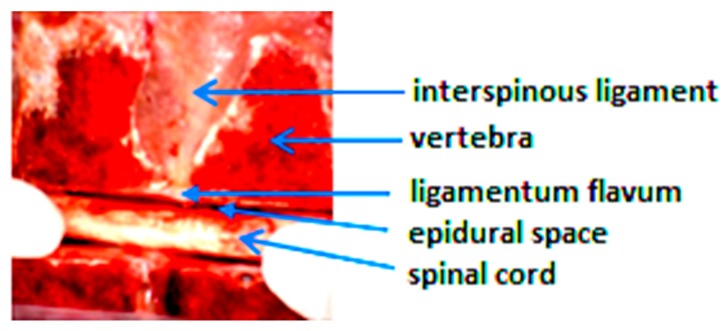
A piece of fresh trunk pork including vertebra. Skin and fat layers are not shown.

A piece of fresh trunk pork including vertebra (L5-L1) was purchased from butcher. It was cut along the middle line of the back bone so that the tissue layers from skin to spinal cord can be explicitly shown in Figure 1. Autofluorescence spectra of the dissected samples of fat, muscle, interspinous ligament (IS), supraspinous ligament (SS) and ligamentum flavum (LF)obtained from the pork were collected by a multimode microplate reader (Infinite 200 Pro, TECAN System, Männedorf, Switzerland. Excitation wavelength was selected for the consequent study according to the autofluorescence spectra of these various tissue samples in order to have the best beneficial for tissue discrimination.

#### 2.2.2. Study Case 2

**Figure 2:**
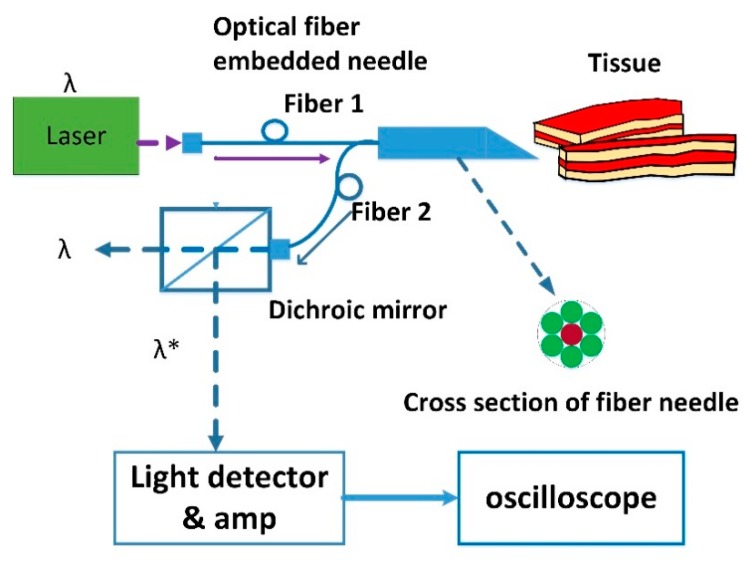
The fiber needle contains seven optical fibers. The one in the center (Fiber 1) which is marked by red color is for light emission. The rest fibers marked in green color are for receiving optical signal from tissues. Laser light with wavelength λ (405 nm) is coupled into Fiber 1. The reflective light from tissue is guided to the dichroic mirror (Delta DCLP425) through Fiber 2. The reflective light is partially transmitted with the same wavelength λ through the mirror into the air and partially reflected by the mirror with fluorescent light of wavelength λ*. The λ* is a band of wavelengths which is greater than wavelength (λ).

The way of obtaining fresh trunk pork was the same as that in the ex vivo study-1. The probing needle is similar to that used in our previous studies [13,14]. A schematic diagram for the experimental setup is shown in Figure 2. The needle used in our studies is a 17-gauge Tuohy needle (Arrow, Morrisville, NC, USA) containing the designed optical stylet. The optical signal from tissue contains wavelengths of primary excitation (λ) and autofluorescence (λ*). Both of which can be separated by a dichroic mirror and are further detected and amplified respectively. The light source was modulated by a 3-Hz clock generated by a control circuit [13]. This light beam was coupled into a single fiber of a fiber bundle, embedded in the stylet needle, by a relay lens [13]. A photodiode has been employed for laser-power monitoring, where a flat reflective mirror has been used for guiding the light beam to the photodiode [13]. The light reflected or backscattered from the tissue was received by a photomultiplier tube (H5783-20, Hamamatsu, Japan) through the rest of the optic fibers of the bundle at the tip of the stylet needle [13]. The signal of photomultiplier tube was amplified and displayed on the screen of an oscilloscope. A personal computer has been used to simultaneously collect and store the data for analysis [13]. The para-vertebral tissues contain skin, fat, interspinous ligament and ligamentumflavum. The tissue was punctured by the fiber needle and only fluorescent light was recorded by an oscilloscope.

### 2.3. Phase Two—In Vivo Studies

A Duroc and Landrance, Chinese native swine with weight of about 30 kg, was used in this study. The animal was intubated and ventilated after induction of general anesthesia, where Tiletamine–zolazepam (5 mg/kg) was given intramuscularly. Anesthesia was then maintained with an intravenous infusion of pentobarbital sodium (15 mg·h^−1^·kg^−1^) for the duration of the studies. The procedure of needle puncture was similar to our previous reports in Reference [13,14] except that only single wavelength light source was employed. The reflective signals (λ and λ*) were recorded by an oscilloscope. All the studies used the same probe and it was disinfected every time an experiment was done.

## 3. Results

### 3.1. Ex Vivo Studies

#### 3.1.1. Study Case 1

**Figure 3:**
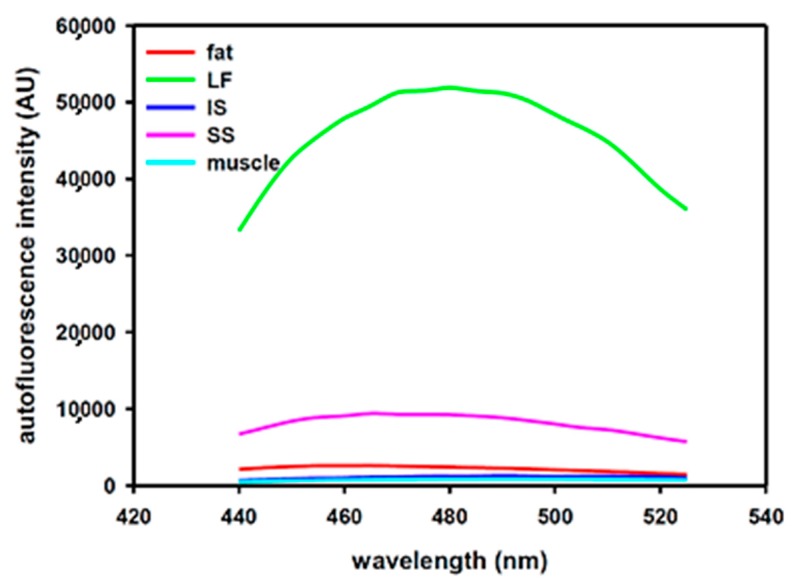
Autofluorescence spectral profiles of fat, interspinous ligament and ligamentum flavum. It should be noticed that the spectra were collected using the microplate reader.

Excitation wavelength 405 nm was selected as the autofluorescence intensity of LF was much higher than those of the other tissues. Figure 3 shows the autofluorescence spectral profiles of these tissues recorded by the multimode microplate reader. In the profiles, the abbreviations of LF, IS and SS represent ligamentumflavum, intraspinous ligament and supraspinous ligament, respectively. The spectral wavelength range was set from 440 nm to 525 nm during recording. The ratio of average magnitude for LF:IS:SS:fat:muscle within the wavelength range is about 58.2:1.4:10.3:2.8:1. This implies that LF has relatively much higher autofluorescence intensity than the other tissues on the way of needle puncture to probe the ES. We used the data obtained from Case 1 as a reference for the Case 2 as the experimental results of Case 1 are from separate tissue layers from skin to epidural space.

#### 3.1.2. Study Case 2

Once the needle is stabbed into tissue and further puncture is carried out on the ex vivo porcine trunk, we can barely see a dim light emitted from the needle tip inside the tissue if the needle is not stabbed too deep. At this moment, the tissue of the needle tip located, unlike the samples obtained from the previous section that can be precisely identified, corresponding to the location in anatomy can only be estimated. As a result, the measured fluorescence intensity in ratio for LF:IS (or SS):fat, which is about 18.5:1.85:1, can be estimated. The results are fairly close to that obtained in the previous section. The data from Case 2 can be used to confirm that our design is feasible by means of the comparison of them with the data from Case 1 as the porcine trunk involved in Case 2 was intact as a whole.

### 3.2. In Vivo Studies

**Figure 4:**
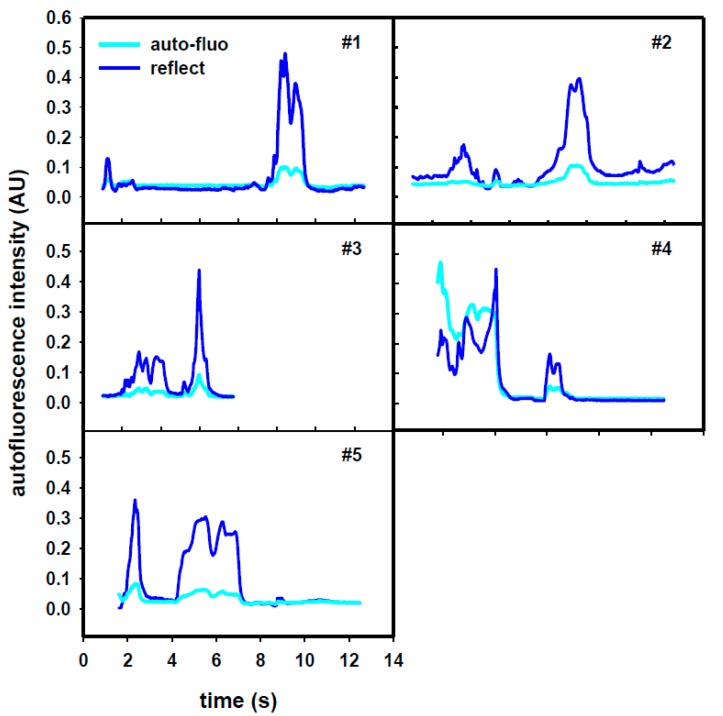
The time-trace profile of needle puncture from skin to epidural space. A total of 5 different paravertebral sites for puncture were recorded. The reflection (Blue) and fluorescence (Cyanate) are simultaneously measured for double assurance of ligamentum flavum detection. The puncture speed is not a constant and consequently the time course should not be expressed as distance or thickness of the tissues.

Figure 4 displays the time-trace profile of needle puncture from skin to epidural space. A total of 5 different paravertebral sites for puncture were recorded. It is noted that the horizontal axis is the time course but the appearance of the signal with respect to the time is needle stabbing speed dependent. In our studies, we kept the needle advancing speed extremely low like general clinical anesthesia procedure in order to not result in accidental puncturing. Since the needle advancing speed has been extremely low, there was ignorable influence by needle movement related to the fluorescence. One can see from the profile that neither the appearance of the double-peak does not occur at the same time slot, nor its widths are the same. Similarly, their amplitudes are also different. In our experiments, the signals of case #1 express the mostly smooth procedure among the 5 ones. It begins with a small peak, which is the reflection from the skin, then moves straight forward to a double peak. In practical meaning, the needle does not hit any obstacle, such as bone, on the way its placement. For case #2, it is similar to case #1. In case #3, the puncture is much faster than the others and only shows a narrower sharp single peak. For case #4, signals resulted from bone hitting creates a very large response which may be dependent on the impact force from the needle or the reflection from the periostem. The cause of relative lower response in both reflectance and autofluorescence, when compared to the others, is unknown. For case #5, the first sharp peak comes from bone hitting. The reason the double peak portion shows a very wide duration is due to that the puncture speed was on purposely slowed down in order to observe the clear M-shape character of the LF in response to the 405-nm excitation. These ES confirmations were all examined by traditional catheter placement and saline injection.

## 4. Discussion

Although LOR is commonly used in humans and reported with an accidental dural puncture rate around 1–2% [18,19], it is not easy to perform in patients with anatomical difficulties such as high body mass index [20], old age [21], spinal deformity [22] and after spine surgery [23]. The incident of accident dural puncture rate in these patients will greatly increase to 6% and even higher [18,19]. For a new operator, especially the resident doctor of anesthesiology, the learning curve for LOR is pretty long [24,25]. Many Investigators have tried to find a new method to replace LOR to help anesthesiologist especially the residents to locate the ES in an easy and reliable way.

Fluorescence (λ*) is a character of some particular biological tissues or cells in response to the stimulated light (λ). According to the ex vivo experiments (Figure 3), we can find that the fluorescent intensity of ligamentumflavum is much higher than those of the fat and interspinous ligament/supraspinous ligament (muscle) for the particular stimulus wavelength (λ). However, the influence of HbO_2_ or Hb in blood should not be neglected as far as in vivo study is concerned. Fortunately, the in vivo study (Figure 4) reveals that the fluorescent signal is simultaneously elevated with the optical signal only on the punctured tissue of ligamentumflavum. The fluorescent signal is barely correlated to the fat and muscle. Compared to the fascia, the intensity of the optical signal is about 3 folds on the ligamentumflavum and the same as the fluorescent one. Therefore, the technique of both reflected and fluorescent lights measurement in double assurance can be achieved.

**Figure 5:**
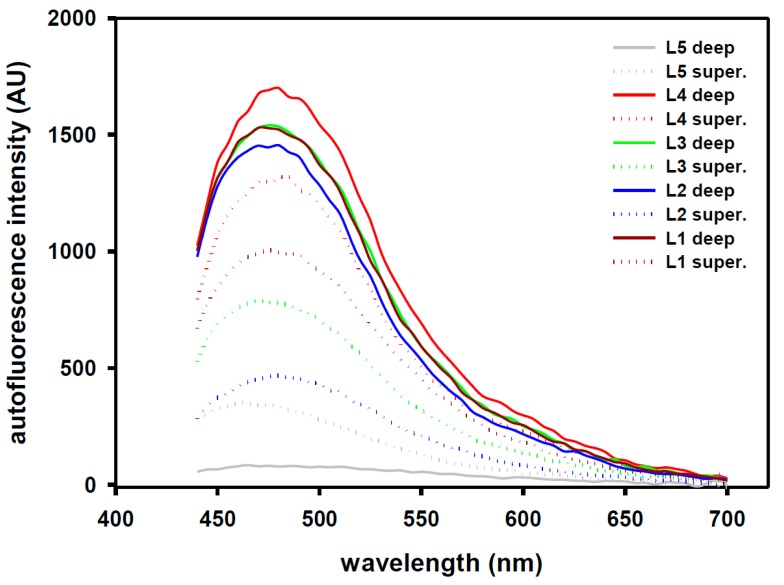
Examined by multimode microplate reader (Infinite 200 Pro, Tecan Group Ltd., Switzerland) to the deep and superficial LF samples that from L1 to L5, 405-nm light was selected as excitation wavelength. The deep component showed higher fluorescence intensity than the superficial one except for the L5.

An interesting question is raised according to the results from our in vivo study, which is the M-shape curve that particularly occurs at the LF for both reflectance and fluorescence signals while needle puncturing. In anatomy, the LF can be separated into superficial LF (faces to ES) and deep LF (connects to interspinous ligament [26]. They are firmly adherent to each other. The superficial component has light yellow color and 2.5–3.5 mm in thickness. On the other hand, the deep component possesses a thin layer of dark yellow structure and with thickness of about 1 mm. Examined by multimode microplate reader (Infinite 200 Pro, TECAN) to the deep and superficial LF samples that from L1 to L5, 405-nm light was selected as excitation wavelength. The deep component shows higher fluorescence intensity than that of the superficial one except for the L5 (see Figure 5).

It is also interesting issues about what the correlation coefficient is between the reflectance and fluorescence whole signals exist throughout the time course and what is it only within the duration of the M-shape or the part of the whole signal with the exclusion of the M-shape. We expected that both signals coming from the tissues, except LF, would be as uncorrelated as possible. Table 1 indicates the correlation coefficients obtained by the statistical method of Spearman Rank Order for the 5 cases mentioned above. In the table, the letter W represents the whole signal, M is for M-shape and W-M indicates whole signal with the exclusion of the M-shape. As indicated in Table 1, the correlations of the duration with M-shape are always higher than those of the other two, particularly to the W-M. This implies that the reflectance and the autofluorescence signals are highly correlated in the region of LF and may not be interfered by the signals from other tissues.

The attenuation of used fiber has been measured for the signal from the fiber. The results including the bend loss are negligible. Both the attenuation and bend loss were used for calibration on the receiver side in order to obtain correct tissue data. The probe is inside the needle, after we reached the ES, the probe was removed and insert the catheter, so the fiber probe will never be used “with” the catheter, instead, they both used “with” the epidural needle. For more verification, we will design the experiments in the future.

## 5. Conclusions

It has been shown that patient positioning, the use of a midline or paramedian approach and the method used for catheter fixation can all influence the success rate for catheter placement [27]. In Reference [27], failure rates of 32% for thoracic and 27% for lumbar epidural were described. In addition to the LOR technique mentioned, ultrasound does not provide adequate resolution to distinguish the tissue layers that the needle travels through or to specifically identify the epidural space and consequently the failure stemming from sonographic-assisted neuraxial placement is still reported [28]. The pressure sensor instantiation in the catheter positioning is the hanging drop technique [27]. As stated in Reference [29], it depends on negative pressure within the epidural space. Recent experimental evidence suggests that negative pressure is poor at reliably detecting the epidural space and if at all, the hanging drop technique is useful only in the sitting position [29]. The OCT has drawn lots of attention recently due to its unbeatable advantage of high imaging resolution [29]. However, the cost of building its system makes it almost impossible to be popularized. In addition, there is still a lack of clinical trials for further evaluation.

In view of the drawbacks, we have presented in this paper a new method to increase the safety of epidural anesthesia. The approach is based on the addition of fluorescence mode to our previously described method based on reflectance spectroscopy. This so-called “double assurance” is able to enhance the specificity for epidural space detection. In this research, the ex vivo and in vivo studies in porcine model of fluorescent responses from the various para-vertebra tissues have demonstrated feasibility to provide valuable information for epidural space detection. It can differentiate between the epidural space and the spinal cord tissue. While combining with our previous technology of reflectance spectroscopy, the procedure in epidural placement with double-assurance provides much safer demand than the traditional techniques. It has been proven that each of the wavelengths we have studied has its advantage. The 405-nm light has been experimentally demonstrated in Reference [30] that it is superior to the others in identifying dense connective tissue which is the major constituent of ligamentum flavum. Despite the advantage of proposed technique, we still have few issues to be addressed prior to realistic clinical scenarios. The first limitation of this study is the ex vivo experiments. In the experiments, the porcine spinal tissues were purchased from a local market. Another concern is that the pigs are common size in our in vivo experiments (the used small piglets weight around 25 kgs). For the 405-nm wavelength, it has been shown recently that it will be affected by red blood cell, blood lipid and hemoglobin Reference [31]. It has also been indicated that the hand shaking can be a variable affecting the puncturing performance [32]. In view of this, we have been paying particular attention to the movement speed and keeping the speed extremely low in order to minimize the effect. Fortunately, the proposed design using 405-nm wavelength can be less affected by the blood flow [33]. In addition, for the results we obtained in Figure 4, there are peaks in the data traces (for some of which there is clear correlation between fluorescence and reflectance), providing potential for false positive results. To address the concern, spectrally-resolved fluorescence measurements will be used. Such techniques are able to provide more specific detection of LF.

Moreover, because the ES detection is based purely on manual observation of the intensity versus time traces in Figure 4. The decision depends on the correlations of the duration with M-shape and correlation coefficients obtained by the statistical method of Spearman Rank Order. More investigations into the decision “threshold” is necessary for clinical applications. In summary, our results show promising steps towards clinical practice. Future human trial is still needed to see if this technology can improve procedure success, reduce complication rates, and/or reduce number of needle puncturing. Efforts are underway to evidence the technique by means of more experiments considering the issues mentioned above. It is believed that by combining 532-nm, 650-nm and 405-nm beams rather than using only single reflectance spectra alone, we can dramatically improve the overall performance.

## Figures and Tables

**Table 1:** Correlation coefficients examined by Spearman Rank Order between reflectance and autofluorescence signals of whole time course duration (W), M-shape only duration (M) and M-shape excluded duration (W-M) for case #1 to case #5.

	Case #1	Case #2	Case #3	Case #4	Case #5
W	0.794	0.939	0.952	0.919	0.903
M	0.995	0.971	0.979	0.978	0.962
W-M	0.674	0.919	0.940	0.879	0.799
